# How do we monitor doxy-PEP impact and when should we consider revisiting guidelines?

**DOI:** 10.1371/journal.pmed.1005276

**Published:** 2026-10-21

**Authors:** Yonatan H. Grad, Rachel Mittelstaedt, Remco P. H. Peters

**Affiliations:** 1 Department of Immunology and Infectious Diseases, Harvard T.H. Chan School of Public Health, Boston, Massachusetts, United States of America; 2 Division of Infectious Diseases, Massachusetts General Hospital, Boston, Massachusetts, United States of America; 3 Department for HIV, Tuberculosis, Hepatitis and Sexually Transmitted Infections, World Health Organization, Geneva, Switzerland

## Abstract

- The 2026 WHO guidelines recommending doxycycline post-exposure prophylaxis (doxy-PEP) represent the first global antibiotic prophylaxis recommendation for bacterial sexually transmitted infections (STIs), with provisions for antimicrobial resistance (AMR) monitoring.
- Doxy-PEP should be governed as an adaptive intervention subject to periodic, transparent review, but establishing decision rules faces challenges.
- Doxy-PEP is expected to select for resistance, including in *Neisseria gonorrhoeae*, *Staphylococcus aureus,* and the gut and skin microbiomes; the scope of clinical consequences is incompletely understood.
- As STI prevalence falls, the benefit per dose declines while the AMR externality per dose is expected to grow, shifting the cost-benefit balance in ways that vary across populations and settings.
- Attributing changes in disease prevalence or resistance patterns to doxy-PEP is methodologically difficult, given incompletely understood and complex drivers of pre-existing trends, surveillance limitations, and heterogeneous uptake, raising questions about study designs, data sources, eligibility criteria, and governance mechanisms.
- A full accounting of benefits and harms is broader than reduced bacterial STI incidence and AMR. Benefits include consideration of patient-centered outcomes such as reduced anxiety regarding STI acquisition, avoidance of repeated treatment visits, and fewer STI-related complications. Harms include drug side effects, such as gastrointestinal adverse effects and concerns about use in pregnancy.

## Abstract 2

In this Policy Forum article, Yonatan Grad and colleagues discuss that while doxy-PEP has shown early success in reducing transmission of bacterial STIs, concerns regarding its contribution to antimicrobial resistance, particularly in N. gonorrhoeae, warrant close monitoring, and discuss why prospective thresholds and adaptive guidelines are needed.

## Introduction

The incidence of bacterial sexually transmitted infections (STIs) has been rising globally for much of the last decade [1]. These infections can potentiate the transmission of HIV, be transmitted from mother to child, and cause other serious sequelae like pelvic inflammatory disease and infertility [2]. Doxycycline post-exposure prophylaxis (doxy-PEP)—a single 200 mg dose of doxycycline taken within 72 hours of condomless sex—is a new intervention to prevent bacterial STIs. Doxycycline has activity against the most prevalent bacterial STI pathogens: *Treponema pallidum* (syphilis), *Chlamydia trachomatis* (chlamydia), and *N. gonorrhoeae* (gonorrhoea). Trials conducted in the US and Europe in men who have sex with men (MSM) and transgender women (TGW), either living with HIV or on HIV pre-exposure prophylaxis (HIV PrEP), established efficacy. Here, doxy-PEP reduced the incidence of chlamydia and syphilis by 70%–85%, and it decreased gonorrhoea incidence by 0%–55%, with variability largely explained by local patterns of gonococcal tetracycline resistance [3–5]. While doxy-PEP is highly effective, there is ongoing uncertainty about the extent to which it will select for antimicrobial resistance (AMR) among both bacterial STIs and other pathogens.

In 2024, the United States Center for Disease Control (CDC) issued guidelines recommending doxy-PEP for MSM/TGW with a recent bacterial STI, with individual patient reassessment recommended every 3–6 months. Guidelines and uptake differ across global settings, with a general approach of adopting doxy-PEP to some extent [6–9], and many countries are working on policy frameworks currently. Even where doxy-PEP is not recommended, as in the Netherlands, broad informal use has been reported [10].

In 2026, the World Health Organization (WHO) published conditional recommendations for doxy-PEP use among MSM/TGW, representing a landmark in public health: the first global recommendation for antibiotic prophylaxis against STIs, together with a provision to monitor AMR [11]. The guidelines mark an advance in STI prevention for MSM/TGW.

The acknowledgment in these guidelines and others of the need to monitor AMR reflects the tension inherent in doxy-PEP [12]: it relies on an antibiotic that exerts ecological pressure across commensal and pathogenic organisms beyond its intended targets. Comparisons are often drawn between HIV PrEP and doxy-PEP, in part because emergence of antiviral resistance was a major concern in the early days of HIV PrEP [13]. Doxy-PEP is unique from HIV PrEP in the broadness of its activity against bacterial bystander pathogens. As doxy-PEP is taken up, we expect—and in some settings are already seeing—declining incidence of syphilis and chlamydia as well as signals suggesting increasing resistance in *N. gonorrhoeae* and bystander organisms [14–18]. But many important questions remain around monitoring and what to do with what we learn. In the case of HIV PrEP, monitoring for resistance in patients who became HIV–positive after taking PrEP was critical to establishing that the benefits of HIV PrEP outweighed the harms at the individual and population scales [13].

Monitoring the impact of doxy-PEP is not straightforward. Surveillance capacity is uneven, attribution of shifts in disease prevalence and resistance to doxy-PEP is difficult, the clinical consequences of changes in resistance prevalence will vary by location, and revising recommendations seen as preventing disease will be hard to enact. These anticipatable difficulties argue for engaging with these challenges now. We suggest that doxy-PEP should be understood as an adaptive intervention that requires continued evaluation and for which modification, when warranted, should be informed by pre-defined criteria.

## Doxy-PEP may shape pathogen-specific dynamics

Syphilis, chlamydia, and gonorrhea are often addressed as a single indication for doxy-PEP, but their resistance dynamics differ. *T. pallidum* has no known acquired resistance to doxycycline, and efforts to develop resistance *in vitro* have suggested that it faces evolutionary roadblocks [19]. The appearance of doxycycline resistance in *T. pallidum* would be a major concern for doxy-PEP, as doxycycline is one of the only alternatives to penicillin treatment. Doxy-PEP is highly effective against *C. trachomatis*, and like *T. pallidum*, no resistance has been observed.

In contrast, *N. gonorrhoeae* develops resistance to tetracyclines, and some areas of the world already have rates of resistance exceeding 80% [20,21]. High-level resistance (Minimum Inhibitory Concentrations ≥ 16 µg/mL) is conferred by the plasmid-borne *tetM*. Evidence of selection for *tetM* in the DOXYVAC study [22], alongside increasing phenotypic resistance among doxy-PEP users in Seattle [17], increasing *tetM* prevalence in the US [14], and an association between decreasing doxy-PEP efficacy against gonorrhea with increasing *tetM* prevalence [16], is consistent with the expectation that widespread antibiotic use will reduce *N. gonorrhoeae* prevalence initially in settings with relatively low starting prevalence of tetracycline resistance, but that it will also select for resistance. As resistant strains spread, effectiveness is reduced. In the absence of changes in behavior or transmission networks, prevalence returns toward prior levels, driven by the resistant strains [15]. The restructuring of the *N. gonorrhoeae* population, in which doxy-PEP could select for strains with reduced susceptibility to cephalosporins, may prove a consequential long-term cost of the intervention [14,23].

The broader ecological costs of doxy-PEP can be seen in the impact on bystander organisms. Among doxy-PEP users, *Staphylococcus aureus* colonization was reduced, but those colonized had higher rates of tetracycline-resistant *S. aureus* [17], with potential consequences of selecting for multidrug-resistant strains [24]. An analysis of the gut microbiome from the DoxyPEP trial found minimal shifts in microbial composition but significant expansion and increased expression of tetracycline resistance genes over six months, with a dose-response pattern [25]. Moreover, a study of 100 mg daily doxycycline for 56 days showed persistent changes to the skin microbiome, even more than 200 days after finishing the antibiotic course [26]. The clinical consequences of these observations are not yet clear.

## The shifting balance of benefits and harms

Changes to the cost-benefit logic of doxy-PEP are anticipatable. As disease prevalence falls, the risk of acquiring an STI per sexual encounter declines. The benefit per dose, measured in infections averted, shrinks accordingly, and the number needed to treat (NNT) to prevent a single infection rises. This is true for any prophylaxis used in a declining epidemic and is an argument for progressively concentrating the intervention on those with the highest residual risk.

Simultaneously, if the increases in AMR result in clinical consequences such as treatment failures and harder-to-treat infections, the AMR externality per dose will grow. This could drive costs that will be borne by future patients facing treatment failures and healthcare systems managing harder-to-treat infections. While those using doxy-PEP gain its benefits now, the costs of resistance would be expected to fall at some point in the future, potentially years later, potentially on others, and where the extent of selection for resistance is only a forecast.

The calculations of benefits and harms will differ across global settings. Where baseline gonorrhea resistance is already very high, where syphilis epidemiology is driven by heterosexual networks, or where the burden of *S. aureus* infections is elevated, the balance of benefits and harms may differ from what was observed in the trial populations. Moreover, in locations without sufficient infrastructure for monitoring disease incidence and antimicrobial resistance, building or expanding this infrastructure adds additional complexity to the cost-benefit calculation.

Benefits and harms will also differ across sexual orientations and gender identities. Doxy-PEP has been highly effective in MSM/TGW, but efficacy in *cis-*gender women has not yet been established. A study of doxy-PEP use in *cis-*gender women in Kenya did not significantly reduce bacterial STIs, although this may have been due to limited use of doxycycline in the intervention arm [27]. Ongoing and planned trials continue to evaluate the efficacy of doxycycline in *cis-*gender women (e.g., NCT06738407and NCT06582966) [28,29]. If such studies conclude that doxycycline prophylaxis is efficacious in *cis-*gender women, consideration will need to be given to expanding eligibility to this group. While STI incidence is lower in *cis-*gender women than in the populations included in the original doxy-PEP studies [4,27], STIs in this group can lead to unique and serious sequelae like congenital syphilis, pelvic inflammatory disease, and infertility [27]. Given the potential severity of the consequences, a higher NNT and associated risk of selection for AMR may be tolerable in *cis-*gender women compared to MSM/TGW.

Benefits and harms may also differ at the individual level. Doxy-PEP is generally well-tolerated, and patients report that it is a relatively straightforward intervention that gives them peace of mind when they have new or multiple sexual partners [4,30]. However, some patients express anxiety about the capacity of doxy-PEP to drive AMR, and others experience side effects like gastritis that may lead to discontinuation [30,31]. If doxy-PEP is recommended for *cis-*gender women, concerns about the safety of doxycycline use in early pregnancy may also lead individuals to discontinue therapy [31].

Analyses about how best to roll out doxy-PEP used assumptions about disease prevalence, effectiveness, and resistance that were guided by data at that time [32]. As these parameters and eligibility criteria change, it will be helpful to revisit these analyses. To do this objectively, this should ideally not be an *ad hoc* action but a scheduled exercise built into governance.

## The challenge of attributing changes to doxy-PEP

Prior to widespread doxy-PEP implementation in the US, syphilis rates were increasing, chlamydia rates were relatively stable, and gonorrhea rates were already declining in some populations for reasons that remain incompletely understood [33]. Similarly, *tetM* prevalence in *N. gonorrhoeae* was rising before doxy-PEP became widely used, plausibly attributable in part to therapeutic doxycycline for chlamydia [14]. These trends complicate before/after comparisons and mean that observed benefits and resistance signals alike cannot be attributed to doxy-PEP without careful analysis.

Surveillance data face additional limitations: changes in representativeness, testing volume, shifts in testing-seeking behavior, reporting lags, and heterogeneous sub-epidemics across geographic and demographic groups all make aggregate trends difficult to interpret. Overestimating the benefit of doxy-PEP leads to continued prescribing with resistance costs, and underestimating the clinical risk of increased resistance leads to passing thresholds at which one might have chosen to act. Similarly, and further underscoring the importance of evaluating doxy-PEP’s impact, overestimating risks and underestimating benefits may result in withholding or failing to expand an effective intervention.

It is worth establishing the study designs and determining the types of data that will be needed to guide inferences around the forces shaping both disease prevalence trends and trends in resistance, including comparisons across settings with different uptake levels and integrated surveillance linking individual doxy-PEP use with STI testing outcomes and resistance data in both pathogens and commensals.

## Clarifying the indirect effects of doxy-PEP

Declining STI prevalence among doxy-PEP users may generate indirect benefits for nonusers through reduced transmission and reduced antibiotic use. One possible example of this indirect effect is the decrease in syphilis among *cis-*gender women recently reported in Seattle King County [34]. Demonstrating a causal link between doxy-PEP prevention of syphilis in MSM/TGW and reductions in rates in *cis-*gender women—which may eventually translate to reduced rates of congenital syphilis—should be a priority.

Conversely, resistance selected among users can spread to nonusers through transmission of resistant organisms and through horizontal transfer of resistance genes in shared commensal environments. The net effect on the broader population is not predetermined and requires ongoing evaluation.

## Monitoring and decision-making

The monitoring provisions in guidelines represent a meaningful advance, but monitoring without pre-specified thresholds for action is surveillance without a decision rule. Several components of an adequate framework should be developed.

What NNT for syphilis prevention would justify reconsidering eligibility criteria? What clinical outcomes of resistance should be tracked? What level of *tetM* prevalence in *N. gonorrhoeae* should trigger guideline review, if any? What about signal in infections with resistant *S. aureus*? If no concerning signal of increased resistance is observed, what might drive considerations of expanding eligibility? Grappling with these questions and determining pre-specified thresholds will enable planning, communication, and consideration of factors that may require flexible responses.

Current STI surveillance was not designed to support the situational management that doxy-PEP guidelines will likely require. Resistance data on *N. gonorrhoeae* globally are available through a few national and international surveillance systems but with geographic limitations and reporting lags. Bystander resistance in *S. aureus* and other organisms is not routinely monitored at population scale, but often through convenience sampling from clinical cases with microbiology data. Even greater challenges arise in tracking clinical outcomes of AMR, including changes in prescribing practices, empiric treatment failures, and infection complications. Integrating AMR surveillance with STI surveillance and prescribing data is technically feasible but not currently operationalized in most settings. While the monitoring framework required for adaptive management is aspirational in high-income countries, it is largely absent in low- and middle-income settings.

Doxycycline is cheap and widely available. Informal use via telehealth platforms, standing prescriptions, or direct acquisition is likely substantial and will continue regardless of guidelines [10]. This means that both stewardship and evaluation are harder than they would be for a more restricted drug, and it suggests we will need to consider new mechanisms for monitoring use.

A formal mechanism, in which the population-level case for doxy-PEP is explicitly reviewed against current epidemiological and resistance data at defined intervals, would provide accountability and a structured opportunity to act. Community advocates have legitimate and necessary roles in this governance process, as their engagement is both ethically required and practically necessary for any policy change to be credible and durable.

## Considerations for next steps

Responses will need to be considered in relation to local epidemiology and resistance profiles. Individual-level off-ramps are embedded in CDC guidance, through reassessment of ongoing need every 3–6 months, accounting for changes in behavior and partnership patterns. The extent to which this happens in practice warrants study.

In case of increasing resistance prevalence, tightening eligibility to prioritize those with the highest baseline risk, defined by recent STI diagnosis, high partner numbers, or network-level exposure during outbreaks, would concentrate the intervention where NNT is lowest and reduce population-level AMR pressure. This is more feasible than broad removal and consistent with evolving stewardship norms. If resistance does not emerge on a scale that raises concern, how should policies adapt?

Pre-specified AMR thresholds for action may also offer a mechanism for policy change. If *S. aureus* resistance to tetracyclines increases more than a pre-specified amount or if *tetM* prevalence in *N. gonorrhoeae* or co-selection for cephalosporin resistance exceeds a defined threshold, guideline review can be triggered rather than awaited. Agreeing on these thresholds prospectively is considerably easier than negotiating them reactively under clinical and political pressure.

The emergence of new tools for managing STIs may also impact the positioning of doxy-PEP in STI prevention. More accessible STI testing, including self-testing and point-of-care diagnostics, can enable earlier treatment and reduction of transmission [35]. Similarly, vaccines for the bacterial STIs would likely have a large impact in reshaping considerations around doxy-PEP [36].

## Conclusions

Doxy-PEP represents a public health achievement. The challenge now is to determine the best course for monitoring and what to do when monitoring detects the changes that theory and early evidence predict. Disease prevalence will fall in some settings, resistance may rise, and the cost-benefit balance could shift. Treating doxy-PEP as an adaptive intervention, one whose continuation requires periodic justification and whose modification is governed by transparent criteria set before they are needed, is more likely to achieve the best outcomes and to sustain public trust than either an indefinite expansion or an unplanned retreat.

## Abbreviations

AMR: antimicrobial resistance
CDC: Center for Disease Control
doxy-PEP: Doxycycline post-exposure prophylaxis
HIV PrEP: HIV pre-exposure prophylaxis
MSM: men who have sex with men
NNT: number needed to treat
STIs: sexually transmitted infections
TGW: transgender women
WHO: World Health Organization.
